# Intestinal Flora as a Potential Strategy to Fight SARS-CoV-2 Infection

**DOI:** 10.3389/fmicb.2020.01388

**Published:** 2020-06-09

**Authors:** Li-Hong He, Long-Fei Ren, Jun-Feng Li, Yong-Na Wu, Xun Li, Lei Zhang

**Affiliations:** ^1^The First Clinical Medical College, Lanzhou University, Lanzhou, China; ^2^Department of General Surgery, The First Hospital of Lanzhou University, Lanzhou, China; ^3^Key Laboratory of Biological Therapy and Regenerative Medicine Transformation Gansu Province, Lanzhou, China; ^4^The Department of Infectious Diseases, The First Hospital of Lanzhou University, Lanzhou, China

**Keywords:** COVID-19, SARS-CoV-2, intestinal flora, gut-lung axis, immunity, SCFAs

## Abstract

The coronavirus disease 2019 (COVID-19), caused by severe acute respiratory syndrome coronavirus 2 (SARS-CoV-2), has spread rapidly worldwide, seriously endangering human health. In addition to the typical symptoms of pulmonary infection, patients with COVID-19 have been reported to have gastrointestinal symptoms and/or intestinal flora dysbiosis. It is known that a healthy intestinal flora is closely related to the maintenance of pulmonary and systemic health by regulating the host immune homeostasis. Role of the “gut-lung axis” has also been well-articulated. This review provides a novel suggestion that intestinal flora may be one of the mediators of the gastrointestinal responses and abnormal immune responses in hosts caused by SARS-CoV-2; improving the composition of intestinal flora and the proportion of its metabolites through probiotics, and personalized diet could be a potential strategy to prevent and treat COVID-19. More clinical and evidence-based medical trials may be initiated to determine the strategy.

## Introduction

Coronavirus disease 2019 (COVID-19), caused by severe acute respiratory syndrome coronavirus 2 (SARS-CoV-2), was first reported in December 2019 (Wu et al., 2020). Since then, the disease has spread rapidly worldwide and has been declared a global pandemic by the World Health Organization. As of 25 May 2020, there were 5,307,298 confirmed cases, including 342,070 deaths (WHO, 2020; Zhu et al., 2020). Considering its strong infectivity, poor prognosis, and lack of effective/targeted drugs, potential prevention and treatment strategies for COVID-19 need to be urgently developed. The damage to host immune defense and the “cytokine storm,” an excessive production of inflammatory cytokines, are believed to be the critical causes of deteriorated health and even death of patients with COVID-19 (Zumla et al., 2016; Pedersen and Ho, 2020; Ye et al., 2020). Given the crucial role played by intestinal flora and its metabolites in regulating immune and inflammatory response of the host, the prospect of modulating intestinal flora for preventing and treating COVID-19 and related illnesses (e.g., viral and/or bacterial pneumonia, acute respiratory infections, or influenza) has attracted considerable attention from the scientific community (Belkaid and Harrison, 2017; Dang and Marsland, 2019; Xu et al., 2020). In order to develop a potential strategy for COVID-19 prevention and treatment by targeting the intestinal flora, we focused mainly on the effects of SARS-CoV-2 on the host intestinal microecology, as well as the possible mechanisms through which intestinal flora regulate immune and inflammatory responses in patients with COVID-19 and related diseases. Particularly, the role of “gut-lung axis” and some indirect evidence for the effect of intestinal flora on the prevention and treatment of COVID-19 have been highlighted here.

## Clinical Manifestations and the Possible Mechanism of Intestinal Microecology Disorders In Patients With COVID-19

The main clinical manifestations of COVID-19 are fever, cough, and acute respiratory distress syndrome. However, gastrointestinal symptoms, such as diarrhea, nausea and vomiting, abdominal pain, and loss of appetite have been reported in an increasing number of COVID-19 patients (Cholankeril et al., 2020; Goyal et al., 2020; Guan et al., 2020; Lin et al., 2020). Interestingly, the COVID-19 patients with gastrointestinal symptoms had more severe disease and these symptoms could be used to predict the development of severe respiratory disorders (Gou et al., 2020; Wan et al., 2020). It is noteworthy that some COVID-19 patients also showed microbial dysbiosis with decreased levels of *Lactobacillus* and *Bifidobacterium* (Xu et al., 2020); the abundance of *Clostridium hathewayi, Clostridium ramosum*, and *Coprobacillus* was positively correlated while that of *Faecalibacterium prausnitzii* was inversely correlated with the severity of the disease (Zuo et al., 2020).

SARS-CoV-2 can identify and invade human cells through the interaction of spike proteins with human angiotensin-converting enzyme 2 (ACE2) (Wu et al., 2020). ACE2 is expressed not only in the lung tissue but also on esophageal and intestinal epithelium; this is the basis of SARS-CoV-2 attacking the digestive tract of the host and leading to intestinal flora dysbiosis and gastrointestinal symptoms (Guan et al., 2020; Holshue et al., 2020; Li M. Y. et al., 2020). Moreover, some studies have reported that SARS-CoV-2 and its nucleic acid were isolated from stool samples of patients with diarrhea (Lamers et al., 2020; Zhou et al., 2020; Zou et al., 2020). These evidences suggest that SARS-CoV-2 may be harbored in the digestive tract of patients and transmitted via the fecal-oral route, affecting the health of the gastrointestinal tract and intestinal flora.

ACE2 is a negative regulator of renin-angiotensin system and is critical for maintaining the homeostasis of blood pressure and the balance of salts and fluid; and ACE2 has local regulatory effects in the pathological changes in several organs, including the heart, kidneys, and lungs (Patel et al., 2017). The association between intestinal flora and ACE2 has also been reported previously: deficiency of ACE2 caused critical impairment of local tryptophan homeostasis in a mouse model, which could alter the intestinal microbiome and susceptibility to inflammation (Hashimoto et al., 2012). ACE2 can also regulate the absorption of nutrients by binding with amino acid transporters on intestinal epithelial cells, which suggests that SARS-CoV-2 might compete with protein nutrients and interfere in their absorption through ACE2 on the intestinal epithelium (Singer et al., 2012; Vuille-Dit-Bille et al., 2015; Javed and Broer, 2019). Cole-Jeffrey et al. indicated that the protective actions of ACE2 against cardiopulmonary disorders could be mediated by its actions on the gastrointestinal tract and intestinal flora (Cole-Jeffrey et al., 2015). A recent study also reported that some specific intestinal microorganisms that can downregulate ACE2 expression in murine gut, such as *Bacteroides thetaiotaomicron, Bacteroides dorei*, and *Bacteroides massiliensis*, correlated inversely with the SARS-CoV-2 load in patient's fecal samples (Zuo et al., 2020). It is known that a healthy intestinal flora plays a vital role in maintaining immune homeostasis and gastrointestinal tract health of the host (Lynch and Pedersen, 2016; Shi et al., 2017). It could be speculated that gastrointestinal symptoms and the changes in immune homeostasis induced by SARS-CoV-2 might be mediated, in part, by the intestinal flora. There could be a potential strategy to fight SARS-CoV-2 infection by targeting intestinal flora.

## Intestinal Flora and the GUT-LUNG Axis

Intestinal flora widely affects host health and is highly correlated with a variety of illnesses, including metabolic, digestive system, and even the respiratory diseases. As shown by 16S rRNA and metagenomics sequencing, the human intestinal flora contains more than 1,000 different microbial species, including bacteria, fungi, and viruses (Grice and Segre, 2012; Sender et al., 2016). On average, each host contains about 160 dominant bacterial species, depending on genetics, environmental factors, and dietary habits (Eckburg et al., 2005; Voreades et al., 2014; Zhernakova et al., 2016). The human intestinal microbiome is a highly dynamic microecosystem and interacts with the immune system. Immune cells induced by a variety of antigens can move between the gut and the lungs through the lymphatic system and/or blood, resulting in the regulation of immune response of both organs. The cross-talk between intestinal and pulmonary tissues mediated by the microbiome and immune cells is called the “gut–lung axis” (Mcghee and Fujihashi, 2012; Date et al., 2017; Ipci et al., 2017).

Many studies have reported that disorders of the intestinal flora were related to lung diseases and respiratory tract infections (Hand et al., 2016; Belkaid and Harrison, 2017; Selber-Hnatiw et al., 2017; Gong et al., 2018; Schirmer et al., 2018). In the mouse model, the depletion of sensitive intestinal bacteria (e.g., *Bifidobacteria*) after neomycin administration increased the susceptibility of the mice to influenza virus infection and pulmonary allergic inflammation (Dharmage et al., 2015; Metsala et al., 2015; Pang et al., 2018); and a recent study reported that the gut population of endogenous *Bifidobacterium* increased to enhance the hosts' resistance to influenza when a lethal influenza infection occurred (Zhang et al., 2020). Bradley et al. showed that the abundance of segmented filamentous bacteria could stimulate the migration of Th17 cells to the lung, augmenting the autoimmune response and aggravating pulmonary lesions (Bradley et al., 2017). Moreover, the intestinal flora and its metabolites, such as short-chain fatty acids (SCFAs) and lipopolysaccharides (LPS), are parts of the intestinal mucosal immune barrier and maintain their normal functions during respiratory tract infections (Leblanc et al., 2017; Sittipo et al., 2019; Visconti et al., 2019). The mucosal immune barrier provides protection against thousands of microorganisms and environmental antigens, and is closely related to the systemic and pulmonary immune function of the host (Abt et al., 2012; Abrahamsson et al., 2014; Donaldson et al., 2016). If the intestinal mucosal immune barrier is disrupted, invading organisms are able to enter the blood or lungs and this could result in septicaemia and acute respiratory distress syndrome (Dickson et al., 2016).

Interestingly, Changes in the pulmonary microenvironment (e.g., by influenza virus or SARS-CoV-2 infection) can also alter the structure and function of intestinal flora (Budden et al., 2017; Dang and Marsland, 2019). In the mouse model, influenza virus infection of the respiratory tract increased the number of Enterobacteria in the intestinal flora while decreasing the number of *Lactobacillus* and *Lactococcus* (Looft and Allen, 2012; Tirone et al., 2019). Similarly, LPS injection in mice lungs resulted in an imbalance of pulmonary microbiota that was accompanied by an intestinal microbiota disbalance, which was caused by bacteria that entered the blood and intestinal mucosa coming from the lung tissue (Sze et al., 2014; Hanada et al., 2018). As mentioned above, many COVID-19 patients have also been reported to present apparent microbial dysbiosis; and intestinal flora alterations were associated with COVID-19 susceptibility and severity (Xu et al., 2020; Zuo et al., 2020).

**Figure 1:**
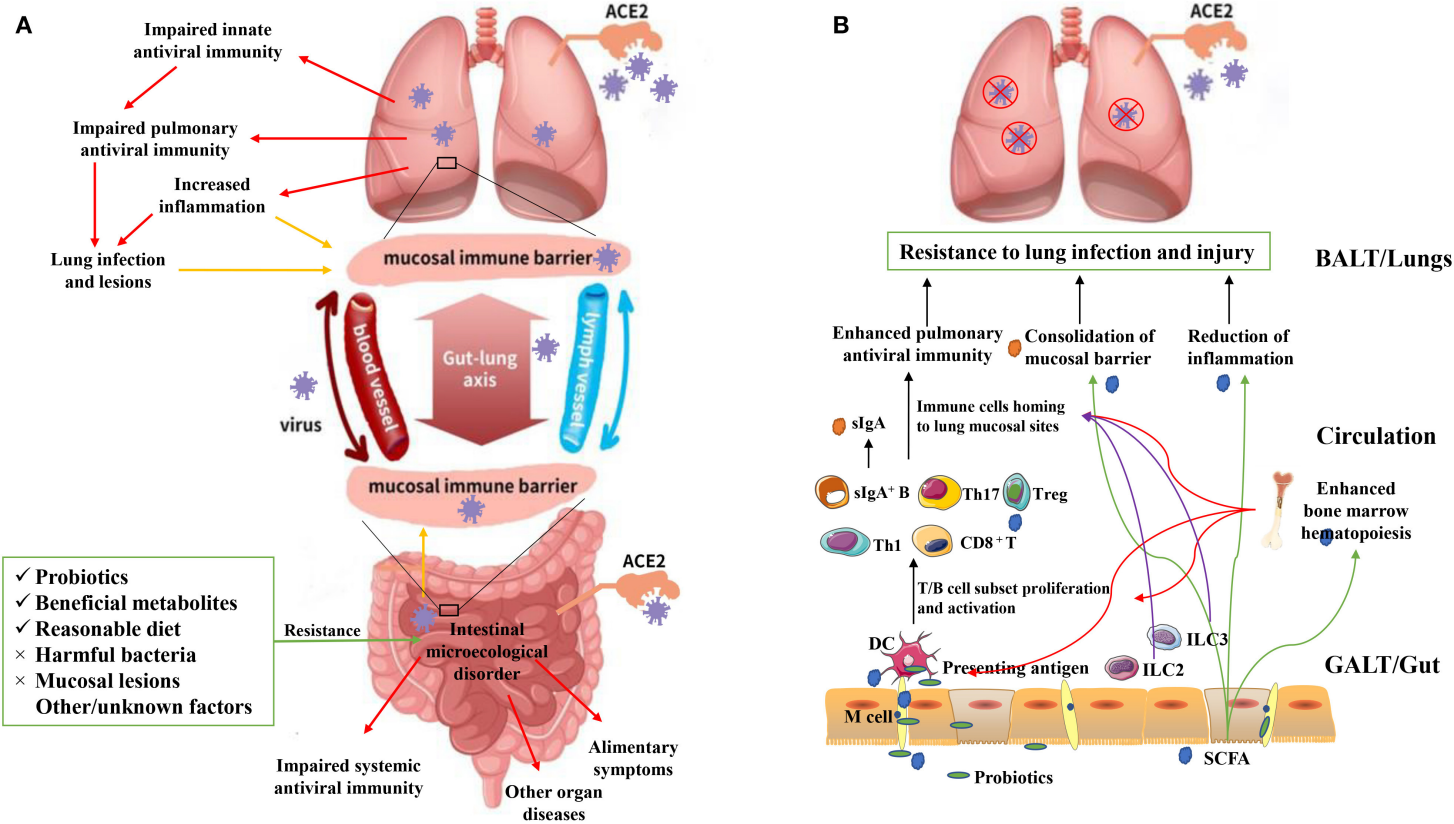
**(A)** Adverse effects of SARS-CoV-2 on the human lungs and intestine. SARS-CoV-2 can be transmitted through the respiratory or digestive tract, directly infecting the host by binding with the ACE2 receptor of pulmonary epithelial or intestinal epithelial cells. This leads to lung and/or intestinal tissue damage and a systemic immune response. However, after initially infecting the lungs, SARS-CoV-2 can break through the mucosal immune barrier and indirectly affect the intestine along the “gut-lung axis”, and vice versa. Intestinal tissue damage, an excessive inflammatory response, and a dysfunctional immune response can lead to intestinal microecological disorder. However, some interventions (e.g., probiotics, beneficial metabolites, and eliminating harmful bacteria) can provide resistance against these adverse effects. The “gut-lung axis” refers to the cross-talk between these two mucosal parts of the human body, which may take place via blood and lymphatic circulation. The yellow arrows represent “increased barrier dysfunction.” The red arrows represent “adverse effects of SARS-CoV-2 infection.” **(B)** Possible model for using probiotics and beneficial metabolites (e.g., short chain fatty acids; SCFAs) against lung infection and injury. Probiotics and metabolites such as SCFAs can be taken up by M cells and presented to T cells as antigens via dendritic cells, leading to T/B cell proliferation and activation. Guided by immune mediators, immune cells are then localized at the lung infection site, enhancing antiviral immunity, and providing protection to the lungs. The intestinal innate lymphoid cells (ILC2 and ILC3) can migrate to the lungs to enhance antiviral immunity via lymphatic and blood circulations (purple arrows). Surface IgA can be produced and transported from gut-associated lymphoid tissues to the surface of the pulmonary mucosa, which can prevent virus adhesion and consolidate the mucosal barrier. SCFAs produced by intestinal flora can be transported to the lungs through the blood, where they can play an anti-inflammatory role and consolidate the lung mucosal barrier (green arrows). SCFAs can also be transported to the bone marrow and enhance its hematopoietic function, further promoting the proliferation and activation of dendritic cells and other immune cells. Overall, these phenomena can enhance the antiviral immunity of the host (red arrows).

To summarize, interaction between the intestinal flora and lungs and ways to promote optimum lung health should be further investigated. Based on the current knowledge regarding the “gut–lung axis,” it can be hypothesized that SARS-CoV-2 not only directly invade human intestinal epithelium cells by being transmitted via the fecal-oral route but also indirectly affect the intestine and intestinal flora along the gut-lung axis, and lung lesions caused by SARS-CoV-2 could potentially be prevented and treated by targeting the intestinal flora (Figure 1A).

## Possible Mechanism Of the Intestinal Flora Regulation of Immune and Inflammatory Responses of the Host

Intestinal flora is supposed to significantly regulate the development and function of the innate and adaptive immune system, tune the immune cells for pro- and anti-inflammatory responses, and maintain immune homeostasis thereby affecting the host susceptibility to various diseases. In case of a pathogenic SARS-CoV-2 infection, a healthy intestinal flora could be essential in maintaining an optimal immune system to prevent excessive inflammatory responses that eventually become detrimental to lungs and vital organ systems. The intestinal flora might regulate host's immune and inflammatory responses along the gut-lung axis, by means of microbial metabolites and the mucosal immune system (Figure 1B).

### Microbial Metabolites

In many microbial metabolites, SCFAs, including butyric acid, acetic acid, and propionic acid, are the most critical metabolites of the intestinal flora. They are extremely important in regulating systemic and pulmonary immune and inflammatory responses (Budden et al., 2017; Goncalves et al., 2018). The most direct function of SCFAs is to reduce the intestinal pH and increase mucin production, which reduces the growth and adhesion of pathogenic microorganisms and improves epithelial integrity, further enhancing the systemic immunity of the host (Fukuda et al., 2011; Jung et al., 2015). SCFAs exert biological effects mainly by inhibiting histone deacetylase (HDAC) and activating G protein–coupled receptors (GPCRs) (Tan et al., 2014; Husted et al., 2017; Li et al., 2018). More specifically, SCFAs can increase the number and function of T regulatory (Treg) cells, T helper (Th) 1 cells, and Th17 effector cells through HDAC inhibition, thus impairing excessive inflammation and immune response in airway diseases along the gut-lung axis (Meijer et al., 2010; Furusawa et al., 2013; Hull et al., 2016; Li et al., 2018). Many studies have shown that GPCRs, especially GPR43, GPR41, and GPR109A, play important roles in the regulation of metabolism, inflammation, and immunity (Den Besten et al., 2013; Kim et al., 2013; Husted et al., 2017; Sun et al., 2017). SCFAs, especially butyrate, have a wide range of anti-inflammatory functions, which are mediated via activation of GPR43 and subsequent activation of β-arrestin 2 by inhibition of the NF-κB pathway (Meijer et al., 2010; Furusawa et al., 2013; Li et al., 2018). SCFAs can also regulate Ly6c(–) patrolling monocyte haematopoiesis and enhance the function of CD8^+^ T cells to confer protection against influenza virus infection through GPR41 activation (Trompette et al., 2018). Butyrate has been reported to induce the differentiation of Treg cells and IL-10/18-producing T cells through GPR109A activation (Singh et al., 2014).

Recent studies have shown that SCFAs represent a link between the bone marrow, gut, and airways (Dang and Marsland, 2019). These molecules have been detected in very small quantities in the lungs, indicating that the lung microbiome does not produce them in large quantities and the circulating SCFAs do not accumulate in the lung tissue. Thus, SCFAs may have a negligible role in the respiratory tract. However, the metabolized intestinal SCFAs can enter the peripheral blood circulation and bone marrow and affect the development of immune cells, which could then be recruited to the lungs and promote lung homeostasis and immunity (Trompette et al., 2014, 2018; Kopf et al., 2015). SCFAs can also promote the generation of progenitors of macrophages and dendritic cells (DCs) in the bone marrow; phagocytic DCs compose the majority of cells that enter the lungs, thus enhancing the function of the T cell subset and triggering a protective mechanism against allergic airway inflammation and respiratory tract infection (Liu et al., 2009; Trompette et al., 2014; Kopf et al., 2015).

In addition to SCFAs, many metabolites of the symbiotic intestinal flora have been reported to be related to host immunity (Rooks and Garrett, 2016). Tryptophan can be used as an energy source by *Lactobacillus* to produce ligands for an aryl hydrocarbon receptor; this receptor is essential not only for the organogenesis of intestinal lymphoid follicles but also for maintaining the homeostasis of the epithelial barrier and intraepithelial lymphocytes (Kiss et al., 2011; Lee et al., 2011; Wynn et al., 2013; Gao et al., 2018). Retinoic acid plays an important role in maintaining intestinal immune homeostasis as it promotes IgA production by B cells and Treg cells development through transforming growth factor β (Kang et al., 2007; Sun et al., 2007; Levy et al., 2016). Niacin has been reported to promote anti-inflammatory properties of colonic macrophages and DCs through GPR109A signaling and to enable them to induce Treg cells and IL-10-producing T cells (Singh et al., 2014). LPS can enhance the mucosal immune response and provide improved resistance against infection by respiratory influenza A virus (Ichinohe et al., 2011). Lactate and pyruvate produced by intestinal bacteria can enhance the immune response by inducing the dendrite protrusion of small intestinal mononuclear cells that express CX3CR1+ via GPR31 signaling (Morita et al., 2019). Desaminotyrosine produced by *Clostridium orbiscindens* has been reported to provide protection against the influenza virus via type I interferon (IFN-1) (Steed et al., 2017).

### Common Mucosal Immune System

The common mucosal immune system (MIS) is an important part of the systemic immunity and forms the first line of defense against infections. It is mainly comprised of mucosa-associated lymphoid tissue, such as gut-associated lymphoid tissues (GALT) and bronchial-associated lymphoid tissue (Mcghee and Fujihashi, 2012). The GALT consist of Peyer's patches (PP), mesenteric lymph nodes (MLN), and numerous lymphocytes scattered in the lamina propria (LP) and intestinal epithelium. The intestinal epithelium also has widely distributed microfold (M) cells (Bekiaris et al., 2014; Brugman et al., 2015). M cells take up antigens from the intestinal mucosa and present them to T cells through DCs, which leads to the proliferation and activation of the T cell subset (Cesta, 2006; Qi et al., 2006). Moreover, the GALT are rich in surface IgA (sIgA) (Mcghee and Fujihashi, 2012). When pathogenic bacteria come in contact with sIgA, they are eliminated, whereas non-pathogenic and beneficial bacteria are not disturbed and remain on the mucosal surface (Bunker et al., 2017; Bunker and Bendelac, 2018). Interestingly, these immune cells and immune factors can be transferred from the GALT to the bronchial-associated lymphoid tissue through blood and lymph vessels (Qi et al., 2006; Samuelson et al., 2015), providing and enhancing resistance to respiratory infections. In a mouse model, activated intestinal group 2 innate lymphoid cells (ILC2s) were found in lungs injected with IL-25, and lungs with pneumonia were reported to have intestinal ILC3s, which could help the body in resisting infection (Huang et al., 2018). In addition, a healthy intestinal flora also plays an important role in regulating Toll-like receptor 7 (a kind of pattern recognition receptor) signal transduction after respiratory influenza virus infection, which has been found to alleviate MIS damage caused by antibiotic treatment in mice (Wu et al., 2013).

Altogether, understanding the possible mechanism of intestinal flora regulating host immune and inflammatory response may provide another preliminary theoretical basis for the prevention and treatment of COVID-19 by targeting intestinal flora. More trials need to be initiated to further study the role played by intestinal microorganisms in regulating the immunity of COVID-19 patients.

## Intestinal Flora-Mediated Enhancement of Antiviral Immunity

Improving the intestinal microecology (e.g., by taking probiotics and beneficial metabolites) (Table 1) may maintain an optimal immune system and prevent an array of excessive inflammation reactions, while also preventing secondary bacterial infections (Levy et al., 2016; Hanada et al., 2018; Descamps et al., 2019). A high-fiber diet was reported to change the proportion of *Firmicutes* and *Bacteroidetes* that could increase the level of SCFAs in the intestine and blood, which in turn reduced the lung damage caused by a respiratory syncytial virus infection. In the mouse model, the same effect was also observed by supplementing acetic acid in drinking water (Meijer et al., 2010; Den Besten et al., 2013; Trompette et al., 2014). The probiotic bacteria of the genus *Lactobacillus* were shown to stimulate respiratory immune responses in mice by increasing inflammatory signals, thereby enhancing the host's defense against respiratory infections (Salva et al., 2010; Yoda et al., 2012). *Lactobacillus casei* enhanced the phagocytic and killing activity of alveolar macrophages and increased IgA, IFN-γ, and TNF-α expression, assisting the host in the fight against influenza virus (Hori et al., 2001). Interestingly, *Bifidobacterium, Lactobacillus paracasei*, and *Lactobacillus rhamnosus* also presented an effect in preventing respiratory infections (e.g., H1N1, H5N1, and H3N2) by enhancing the vaccine response (Lei et al., 2010; Samuelson et al., 2015). Recent studies have shown that traditional Chinese medicine may have beneficial effects on the recovery of patients with COVID-19, possibly by enhancing the intestinal microecological balance to improve immunity (Xu et al., 2017, 2020; Wang et al., 2019). Notably, a pre-published study (as yet, not peer-reviewed) suggested that the risk of developing severe COVID-19 among patients with vitamin D deficiency was significantly higher than in patients with normal vitamin D levels; moreover, vitamin D may reduce COVID-19 severity by suppressing the cytokine storm displayed by COVID-19 patients (Daneshkhah et al., 2020). However, whether this difference is mediated by intestinal flora remains to be further studied. Nonetheless, what we already know is that Vitamins A and D can enhance the intestinal barrier function and mucosal immune response by maintaining the normal function of ILC3s and T cells (Cantorna et al., 2019). Overall, it is apparent that probiotics and diet-mediated modulation of intestinal flora can influence immunity. The administration of personalized probiotics and diet may be thoughtfully considered for COVID-19 patients to accelerate recovery and improve prognosis.

**Table 1:** Changes in the immune and inflammatory response upon administration of different probiotics and products for pulmonary infectious disease treatment.

**Species or products**	**Major physiological effect**
**Microbial metabolites**	
SCFAs	Maintenance of mucosal barrier
	Enhanced antiviral immune reaction
	Anti-inflammatory effect
Retinoic acid	Increased IgA level
	Treg cell development
Niacin	Anti-inflammatory effect
	Increased activity of macrophages and DCs
	Development of Treg cells and IL-10-producing T cells
DAT	Increased IFN-1
**Probiotics**	
*Lactobacillus acidophilus*	Enhanced inflammatory signals
	Enhanced antiviral immune reaction
*Lactobacillus rhamnosus*	Enhanced antiviral immune reaction
	Enhanced vaccine immune efficacy
*Lactobacillus casei*	Enhanced phagocytic and killing activity of alveolar macrophages
	Increased levels of IgA, IFN-γ, and TNF-α
*Bifidobacterium*	Enhanced vaccine immune efficacy
**Others**	
Vitamins A and D	Enhanced intestinal barrier function and mucosal immune response
	Maintenance of the normal function of ILC3s and T cells
High-fiber diet	Increased level of SCFAs

*SCFAs, short-chain fatty acids; Treg cells, T regulatory cells; ILC3s, group 3 innate lymphoid cells; DCs, dendritic cells*.

## Conclusion and Prospects

The COVID-19 pandemic presents a significant social and economic burden worldwide, and studies on the disease, unfortunately, remain at a very preliminary stage. This review provides a novel suggestion that the intestinal flora may partially mediate the effects of SARS-CoV-2 on the both local gastrointestinal response and systemic immune response of the host, and thus be a target for COVID-19 prevention and treatment. This is consistent with emerging data, which suggest that the gut microbiota plays a key role in predicting the blood proteomic biomarkers that determine the abnormal inflammatory state of individuals with severe COVID-19 (Gou et al., 2020). Moreover, considering the fact that elderly people have less diverse intestinal flora in which beneficial microorganisms (e.g., *Bifidobacterium*) lose ground (Nagpal et al., 2018), this is in line with the observation that older individuals are more susceptible to SARS-CoV-2 and more severe COVID-19 (Goyal et al., 2020; Lake, 2020). The structure and function of the intestinal flora could be a potential biological mechanism behind the diverse susceptibility of different groups of people to SARS-CoV-2. However, the specific mechanisms through which the SARS-CoV-2 infection influences the intestinal microbial community among patients of different ages, ethnic groups, and geographical locations remain to be seen.

The prevention and treatment strategies for SARS-CoV-2 infection considering gastroenterology and intestinal microbiota have received great attention (Gou et al., 2020; Li L. Y. et al., 2020). In February 2020, China's National Health Commission and National Administration of Traditional Chinese Medicine suggested the use of probiotics in patients with severe COVID-19, which has shown good efficacy (National Health Committee of the People's Republic of China, 2020). As shown by existing indirect evidences, there is a potential strategy to prevent and treat COVID-19 through the improvement of intestinal flora composition and of its metabolites. This could be performed using probiotics, personalized diet, and traditional Chinese medicine to balance the immune function and suppress “cytokine storm.” Some specific intestinal microorganisms that can downregulate intestinal ACE2 expression has also been considered as the potential target to fight against SARS-CoV-2 (Zuo et al., 2020). These insights will add new dimensions to understanding SARS-CoV-2 and COVID-19, and can also be helpful for designing a more reasonable and personalized treatment plan for patients, which would be of great significance for assigning medical resources.

It is worth noting that the rationale for using probiotics in COVID-19 is derived from indirect evidence. Different kinds of probiotics and/or different doses of probiotics usually have different biological effects on the host. To further understand the specific mechanism and elucidate the benefits of personalized functional food including probiotics and metabolite products in the prevention and treatment of COVID-19, more clinical trials and evidence-based medical data are needed.

## Author Contributions

All authors contributed to the critical analysis of the collected data and writing the manuscript, and all authors approved the final manuscript.

## Conflict of Interest

The authors declare that the research was conducted in the absence of any commercial or financial relationships that could be construed as a potential conflict of interest.
